# Evaluation of road safety policies and their enforcement in Mexico City, 2015–2019: an interrupted time-series study

**DOI:** 10.1136/ip-2022-044590

**Published:** 2022-09-12

**Authors:** Carolina Quintero Valverde, Carolina Perez-Ferrer, Luis Chías Becerril, Armando Martínez Santiago, Héctor Reséndiz Lopez, Javier Prado Galbarro, D. Alex Quistberg, Ana V Diez Roux, Tonatiuh Barrientos-Gutierrez

**Affiliations:** 1 Center for Research in Nutrition and Health, National Institute of Public Health, Cuernavaca, Mexico; 2 Programa Investigadores por México, National Council of Science and Technology (CONACYT), Mexico City, Mexico; 3 Institute of Geography, National Autonomous University of Mexico (UNAM), Mexico City, Mexico; 4 Center for Research in Population Health, National Institute of Public Health, Cuernavaca, Mexico; 5 Urban Health Collaborative, Drexel University, Philadelphia, Pennsylvania, USA; 6 Environmental & Occupational Health, Drexel University, Philadelphia, Pennsylvania, USA; 7 Department of Epidemiology & Biostatistics, Drexel University, Philadelphia, Pennsylvania, USA

**Keywords:** Legislation, Public Health, Motor vehicle � Occupant, Epidemiology, Process/impact evaluation, Mortality

## Abstract

**Background:**

Mexico City approved new road safety policies in 2015, which included lower speed limits and higher fines for traffic offences. In 2019, economic fines were replaced by a point penalty system among other changes. This study evaluates these policies on road traffic collisions, injuries and deaths.

**Methods:**

Collisions data came from insurance collision claims (January 2015 to December 2019) and road traffic deaths from vital registrations (January 2013 to December 2019). We conducted an interrupted time series analysis for each outcome using negative binomial regression models with an offset of insured vehicles (collisions) or total population (deaths). Then, we classified the 16 municipalities in the city into enforcement and no-enforcement groups based on presence or absence of automated traffic enforcement devices and conducted a controlled interrupted time series analysis.

**Results:**

The 2015 road safety policies had no effect on total collisions and collisions resulting in injury but were associated with a 0.2% (95% CI −0.3 to 0.0) decline in the mortality trend. The 2019 policies had no effect on total collisions but were associated with a 1.5% increase in the trend of collisions resulting in injuries and with a 2.7% (95% CI 1.0 to 4.5) increase in the mortality trend. Postpolicy trends in enforcement versus no-enforcement municipalities were not significantly different.

**Conclusion:**

Policies that included high economic penalties for speeding and dangerous behaviours were effective in decreasing traffic mortality while removing economic penalties and replacing them with a point penalty system were associated with an increase in collisions, resulting in injury and mortality.

**WHAT IS ALREADY KNOWN ON THIS TOPIC**

- Effective road safety policies must be evidence based, enforced and include clear, timely and unbiased penalties when violations occur.
- Mexico City adopted new road safety policies in 2015 and 2019, which have not been formally evaluated.

**WHAT THIS STUDY ADDS**

- This study leverages two independent data sources, insurance claims and mortality data, to evaluate road safety policies implemented in Mexico City using a quasi-experimental design.
- A package of lower speed limits and higher fines for traffic offences was associated with lower mortality, while replacing economic fines with a point penalty system was associated with an increase in severe collisions and deaths.

**HOW THIS STUDY MIGHT AFFECT RESEARCH, PRACTICE AND/OR POLICY**

- This study supports economic penalties to enforce speed limits and other road safety regulations. It shows that a point penalty system leading to civic education courses was detrimental to health, potentially because of difficulties in its implementation.

## Introduction

Traffic fatalities account for 1.25 million annual deaths and 50 million injured people around the world, with over 90% of deaths occurring in low-income and middle-income countries.^1^ They are the main cause of death among people between 5 and 29 years old, and the eighth cause of death for all age groups.^1^ The WHO recommends that countries enact and enforce legislation on five key risk factors for road traffic deaths and injuries: speeding, drink-driving, use of motorcycle helmets, use of seat belts and use of child restraints.^2^ Effective road safety policies must be evidence based, enforced well and include clear, timely and unbiased penalties when violations occur.^3 4^

Mexico has an unacceptably high road traffic mortality rate, which features among the first causes of death, especially among young people.^5^ In 2011, the country designed and adopted a National Road Safety Strategy,^6^ based on the Decade of Action for Road Safety 2011–2020. However, federal road safety laws did not exist up until 2022 when a new General Law of Mobility and Road Safety was approved.^7^ Before 2022, each municipality regulated their roads as they deemed appropriate. As such, results of the strategy have been highly heterogeneous across the country with some states far from meeting the goal of reducing road traffic mortality by 50%.^8^ Mexico City is among the states that was not on course to achieve the goal by 2020.^5 8^

In Mexico City, new road traffic policies came into effect on 15 December 2015,^9^ framed under Vision Zero^10^ and the National Road Safety Strategy.^6 10^ The policies focused on reducing speed and increasing fines for traffic violations and were paired with automated enforcement devices that were installed in some Mexico City municipalities.^9^ In 2019, economic fines for speeding and other traffic violations were substituted by a point penalty system, leading to mandatory road safety courses and social service. None of these changes have been formally evaluated, thus, to date, their impact on road safety outcomes is unknown.

The objective of this study was to estimate the impact of the road safety policies implemented in December 2015 and in June 2019 on total collisions, collisions resulting in injury and road traffic mortality in Mexico City. We hypothesised that total collisions, collisions resulting in injury and road traffic mortality would decline after December 2015 and that the decline would be more pronounced in municipalities with automated traffic enforcement devices compared with those that did not have them.^11–13^ In terms of the 2019 changes, we hypothesised that collisions, collisions resulting in injury and mortality would increase because economic fines were removed.^3 14^

## Methods

### Policy characteristics

The 2015 policy included (1) lower speed limits, introduction of automated photo enforcement of speed (subsequently referred to as speed cameras) and higher fines for speeding vehicles and (2) traffic enforcement cameras to detect nine motoring offences (see box 1).

Box 1 **Nine dangerous behaviours detected by automated enforcement cameras**

1. Not using a seatbelt (for all passengers).
2. Prohibited turns.
3. Invasion of exclusive bus lane (bus rapid transit).
4. Invasion of bicycle lane.
5. Wrong-way driving.
6. Invasion of pedestrian crossing during red traffic lights.
7. Not stopping at red lights.
8. Distracting behaviours (eg, cell phone use).
9. Driving with children in front seats.

The new speed limits were: 80 km/hour in freeways with grade-separated road junctions, 50 km/hour in arterials, 40 km/hour in collectors and 20 km/hour in school zones.^9^ Sixty-six automated traffic enforcement devices (including overt speed cameras) were installed across the city, which were linked to the new fines for motoring offences (see online supplemental file 1, figure 1).

10.1136/ip-2022-044590.supp1 Supplementary data

**Figure 1:**
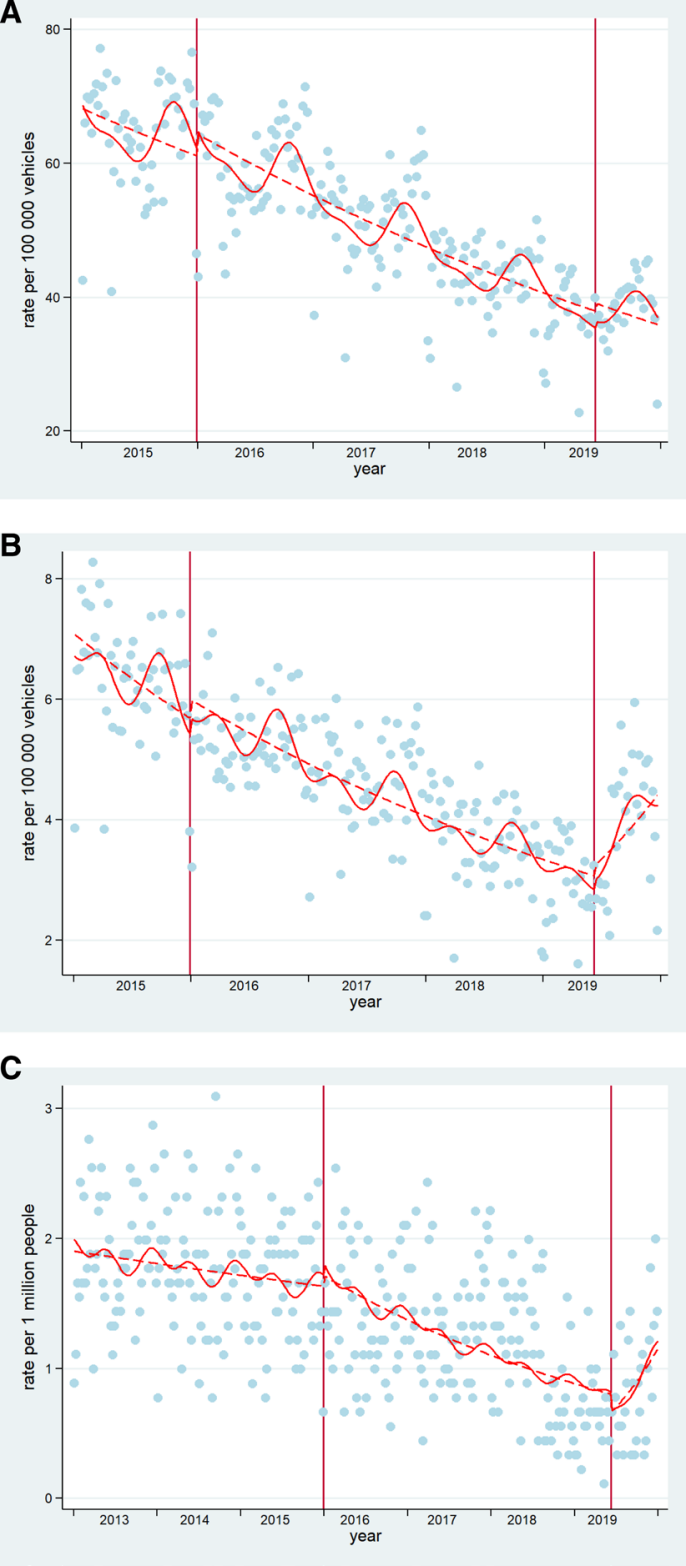
Interrupted time series of (A) total collision rate, (B) collision resulting in injury and (C) mortality rates in Mexico City. Blue dots=cases presented in Mexico City. Dashed lines=deseasonalised trends (Note for C: seasonality did not affect mortality trends, hence no adjustment). Continuous lines=trends. Vertical lines: mark the policy in December 2015 and June 2019. These are original figures created by the research team.

The 2019 policy removed economic penalties for speeding and the nine motoring offences (box 1) for vehicles with Mexico City number plates, except for invasion of the exclusive bus lane and speeding detected by handheld devices; this change occurred on 1 January 2019. Then on 8 June 2019, a point penalty system that leads to mandatory courses and social service was introduced to replace economic penalties. There were no changes to the speed limits. Additionally, some of the existing automated traffic enforcement devices were relocated, new ones were installed and preventive speed monitors (signs that show current speed) were installed around schools.

### Data sources

To assess the effect of the 2015 and 2019 road safety policies, three outcomes were evaluated: weekly total collisions, weekly collisions resulting in injury and weekly deaths from road traffic collisions.

Number of collisions and collisions resulting in injury were obtained from insurance collision claims (AXA Mexico). The data were collected by claims adjusters at the site of the collision using an electronic device. These data were available for public use from January 2015 to December 2019 and include information on the date of the collision, location (coordinates and adjuster reported location), type of vehicle involved and whether there were injuries or deaths.^15^ Data were processed and cleaned, mapping collisions and keeping only those georeferenced within Mexico City boundaries as well as coded to Mexico City in the reported location variable (91.3% of possible records; see online supplemental material for more details, Table S1 and Figure S2). To calculate weekly rates of collisions for Mexico City, we used an estimate of the number of insured vehicles as denominator. The number of registered vehicles per month was obtained from the National Institute for Geography and Statistics (INEGI)^16^ while the proportion of insured vehicles was obtained from the Mexican Association of Insurance Companies.^17^ We used linear interpolation to complete weekly information on insured vehicles using monthly data.

Mortality data were validated and reported by INEGI^18^ from death certificates filed mainly by the Health Sector, using the International Classification of Disease, 10th Revision (ICD-10)^19^ for diagnosis codes. We used data from January 2013 to December 2019 and included deaths with the following ICD-10 codes: V02–V04 (0.1–0.9), V09, V092, V09.3, V09.9, V12–V14 (.3-.9), V19.4–V19.6, V19.9, V20–V28 (0.3–0.9), V29, V30–V39, V40–V79 (0.4–0.9), V80.3–V80.5, V81.1, V82.1, V82.1, V83–V86 (.0-.3), V87–V89.2 y V89.9.^5^ Official total mid-year population estimates from the National Population Council were used to calculate mortality rates for Mexico City and its municipalities for the study period 2013–2019.^20^ We used linear interpolation to complete weekly population data from yearly estimates. Data sets used for analyses are available in a digital repository.^21^

The location of automated traffic enforcement devices was obtained from the Transit Department of Mexico City and was validated using Google street view.

### Statistical analyses

We first described the characteristics and trends of total collisions, collisions resulting in injury and road traffic mortality at the city level from January 2015 to December 2019. We then conducted interrupted time series (ITS) analyses for each policy (2015 or 2019) and outcome separately. Three variables were used in each model: the outcome (Y)—weekly total collisions or collisions resulting in injuries or deaths—the time elapsed since the start of the study in weeks (T) and a dummy variable indicating the policy coded 0 before the policy was implemented and 1 after the policy implementation (X_t_).^22^ Online supplemental table S2 details prepolicy and postpolicy dates and number of data points for each outcome and policy year. As offset in the models, registered insured vehicles or population were included.

The following basic model was tested:

Equation 1.

$$Y_{t}=\beta_{0}+\beta_{1}T+\beta_{2}X_{t}+\beta_{3}TX_{t}$$

where Y_t_ is the outcome at time t, β_0_ represents the baseline level at T=0, β_1_ is interpreted as the change in the outcome associated with a time-unit increase (representing the underlying prepolicy trend), β_2_ is the level change following the 2015 or 2019 policy and β_3_ indicates the slope change following the 2015 or 2019 policy (using the interaction between time centred at the policy, $T-T_{i},$ and policy $X_{t}$ ).^23^ Models for the 2015 policy included from 1 January 2015 (T=0) to 31 December 2018. Models for 2019 policy included from 15 December 2015 (T=0) to 31 December 2019.

As a second step, we conducted a controlled ITS (CITS) analysis at municipality level to investigate whether enforcement modified the impact of the 2015 road safety policy. We compared the time series for each outcome for two groups: enforcement group, defined as municipalities with at least one automated traffic enforcement device (speed camera and/or traffic enforcement cameras) in 2015 and the no-enforcement group, defined as municipalities without automated traffic enforcement devices in 2015. The end date for the controlled data series was December 2018 because economic fines linked to automated traffic enforcement devices were suspended in January 2019 and also because some devices were relocated during the first semester of 2019. In addition to the three variables described in equation 1, we introduce G, which denotes municipalities with enforcement (G=1) or without enforcement (G=0) and the interaction GTX (time * policy * enforcement), which represents the difference in the time slope following the start of the policy in enforcement compared with no-enforcement municipalities. See online supplemental material for equation.^24^

Negative binomial regression models with robust SEs were used because count data were overdispersed. Models were adjusted for seasonality effects using Fourier terms.^22^ All analyses were conducted in STATA V.14.

Four sensitivity analyses were conducted. First, we changed the 2015 policy start date from 15 December 2015 to 1 June 2016 to consider a transition period of 6 months because it may have taken drivers some time to become aware of the changes in the regulations and new fines. Second, we kept only private vehicles for the 2015 analysis (excluding taxis, motorcycles and public transport) on account of two reasons: (A) in practice, the fines for motoring offences detected by automated traffic enforcement devices applied to private vehicles only and (B) automated traffic enforcement devices were mostly located in the central lanes, which are not used by public transport. Third, we adjusted the 2019 policy models by a variable that indicated weeks with gasoline shortages. During the second week of January 2019,^25^ Mexico City suffered gasoline shortages, which could bias our results for the effect of the 2019 changes towards a decrease in our outcomes of interest.

The fourth sensitivity analysis is related to the mortality analysis only. We redistributed ill-defined and partially defined ICD-10 codes to account for a potential underestimation of road traffic deaths due to these ill-defined and partially defined codes.^26^ The redistribution and imputation process were applied to the following codes: Y34 (unspecified event, undetermined intent), Y872 (sequelae of events, undetermined intent), Y899 (sequelae of unspecified external cause), X59 (ill-defined unintentional injuries), V99 (ill-defined transportation injuries) and Y32 (crashing of motor vehicle, undetermined intent). Y34, Y872 and Y899 were distributed among external causes, X59 among unintentional external causes,^27^ V99 among land and other transport injuries and Y32 among land transport injuries. Analyses were repeated with the modified data set.

## Results

### Descriptive analyses

There were 347 259 collisions and 31 678 collisions resulting in injury from January 2015 to December 2019 (table 1). Most collisions involved automobiles (85%) and had a low level of vehicle damage (86%). During the period January 2013 to December 2019, there were 4669 road traffic deaths. The rate of collisions, collisions resulting in injury and mortality decreased over time (table 2).

**Table 1:** Characteristics of collisions registered by AXA insurance in Mexico City, 2015–2019

	Number (n)	Percentage
Total traffic collisions	347 259	100
Collisions resulting in injury	31 678	9.12
Type of vehicle involved		
Automobile	295 054	84.97
Truck	45 453	13.09
Light truck	3 528	1.02
Motorcycle	1 667	0.48
Not available	1 557	0.45
Level of vehicle damage		
Low	164 513	47.37
Medium	5 496	1.58
High	1 001	0.29
No damage*	143 317	41.27
Not reported	32 932	9.48
Deaths due to road traffic collisions†	4 669	100

*Claim adjusters record ‘no damage’ when the collision results in superficial damages such as paint marks or scratches (Personal Communication, Olvera O, AXA Mexico).

†From vital registration data 2013–2019.

**Table 2:** Descriptive statistics for Mexico City in each period of analysis

	January 2015–December 2015	January 2016–December 2018	January 2019–December 2019
Total population, mean	9 059 528*	9 048 157	9 031 213
Number of registered vehicles, mean	4 997 606	5 498 341	6 084 903
Number of insured vehicles, mean	2 548 326	2 673 485	2 819 549
Collision rate (per 100 000 insured vehicles)	3 273.0	2 672.3	1 885.8
Collision resulting in injury rate (per 100 000 insured vehicles)	321.3	235.5	174.9
Mortality rate (per 100 000 inhabitants)	9.2*	6.7	4.1

*2013-2015.

### ITS analyses for Mexico City

Table 3 summarises the effect of the policies on the three outcomes of interest. After controlling for seasonality, the 2015 policies had no effect on total collisions and collisions resulting in injury; there had been a declining trend of both total collisions and collisions, resulting in injury pre-2015 (0.02% and 0.04% weekly declines, respectively), which continued unchanged after the 2015 policies. The 2019 policies had no effect on total collisions but were associated with a 1.5% change (increase) in the trend of collisions, resulting in injury; from a 0.04% weekly decline over the period January 2016 to June 2019 to an increase in 1.1% per week after June 2019 (see figure 1A, B).

**Table 3:** Effect of the 2015 and 2019 traffic enforcement regulations on total collisions, collisions resulting in injury and mortality due to road traffic collisions in Mexico City

	Total collisions			Collisions resulting in injuries			Mortality from road traffic collisions		
	IRR	p	95% CI	IRR	p	95% CI	IRR	p	95% CI
2015									
Step level change 2015	1.057	0.197	0.971 to 1.151	1.031	0.595	0.922 to 1.153	0.960	0.580	0.829 to 1.111
Pre-2015 trend* ^**^	0.998	0.084	0.995 to 1.000	0.996	0.019	0.993 to 0.999	0.999	0.099	0.998 to 1.000
Post-2015 trend† ^**^	0.997	0.000	0.997 to 0.997	0.997	0.000	0.996 to 0.997	0.997	0.000	0.996 to 0.998
Slope difference – 2015‡	0.999	0.536	0.997 to 1.002	1.000	0.795	0.997 to 1.004	0.998	0.038	0.997 to 1.000
2019									
Step level change 2019	1.028	0.600	0.927 to 1.140	1.066	0.435	0.909 to 1.250	0.788	0.133	0.577 to 1.075
Pre-2019 trend§ ^**^	0.997	0.000	0.997 to 0.997	0.996	0.000	0.996 to 0.997	0.996	0.000	0.995 to 0.997
Post-2019 trend¶ ^**^	0.997	0.358	0.991 to 1.003	1.011	0.028	1.001 to 1.021	1.023	0.008	1.006 to 1.041
Slope difference – 2019	1.000	0.973	0.994 to 1.006	1.015	0.003	1.005 to 1.025	1.027	0.002	1.010 to 1.045

*January–December 2015 (mortality January 2013–December 2015).

†January 2016–December 2018.

‡Slope difference pre-post policy quantified as exponentiated interaction coefficient (coefficient β3 in equation 1, refer to methods).

§January 2016–7 June 2019.

¶8 June 2019–31 December 2019.

**Weekly change in the outcome.

In terms of mortality, the 2015 policies were associated with a 0.2% decline in the trend; from a fairly flat prepolicy trend to a 0.3% weekly decline in mortality postpolicy. In contrast, the 2019 policy was associated with a 2.7% increase in the mortality trend. Mortality in the period January 2016 to December 2018 had been declining by 0.4% per week and after the 2019 policy mortality increased by 2.3% per week (see figure 1C).

### CITS analyses for enforcement and control group

**Figure 2:**
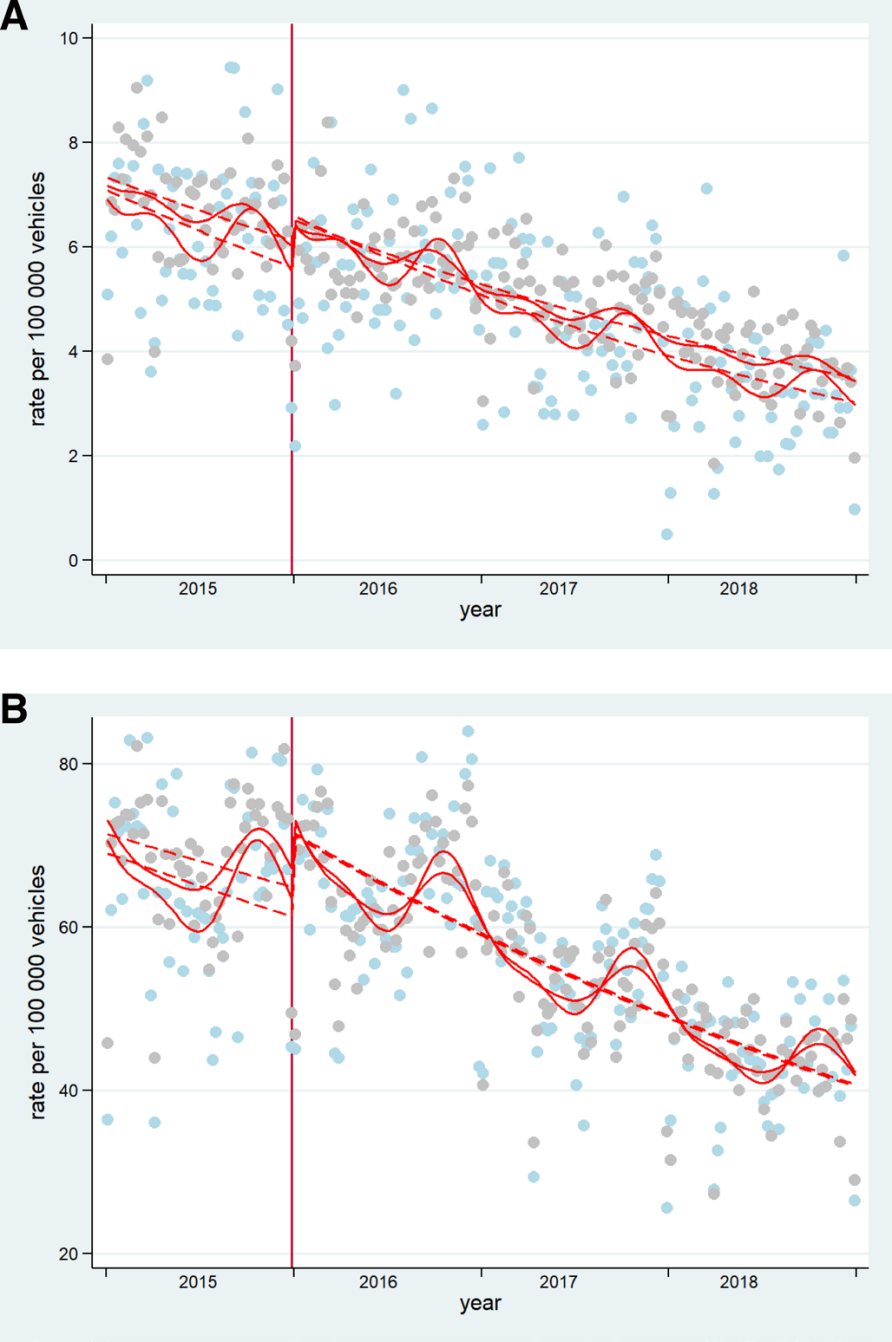
Controlled Interrupted Time Series of A) total collision rate, B) collision resulting in injury in control and enforcement groups (respectively). Gray dots= cases presented in enforcement group. Light blue dots=cases presented in control group. Dashed lines=deseasonalized trends. Vertical lines: mark the policy in 2015. These are original figures created by the research team.

Online supplemental table 3 presents the results of the analyses, which explored enforcement as an effect modifier of the 2015 policies. After controlling for seasonality, the CITS analysis showed that there were no differences between enforcement and no-enforcement municipalities in the postpolicy trend of total collisions and collisions resulting in injury (see figure 2A, B) The model for mortality did not fulfil the assumptions needed for the correct interpretation of CITS, that is, parallel prepolicy trends between control and enforcement groups.

### Sensitivity analysis

The main results for the effect of the 2015 policy on total collisions and collisions resulting in injury were not affected by changing the policy date from December 2015 to June 2016, nor when only collisions involving private vehicles were included in the analyses (see online supplemental file tables S4 and S5. When the data set with redistributed ill-defined and partially-defined mortality codes was used for analyses, the effect of the 2015 policy on mortality was no longer statistically significant. However, the direction of the effect (decline in mortality) was consistent with the main analyses (see online supplemental table 6 and figure S3).

The main results for the effect of the 2019 policy on collisions were not affected when we adjusted for gasoline shortage. In terms of the effect on mortality, the ill-defined and partially defined code redistribution significantly attenuated the effect of the 2019 policy although the direction of the association (increase in mortality) was consistent with the main analyses (see online supplemental figure S3).

## Discussion

Using a quasi-experimental design, this study found that the 2015 road safety policies involving lower speed limits, higher fines and automated traffic enforcement devices were associated with a decrease in road traffic mortality. The 2019 changes, which included replacing economic fines with a penalty point system and community service, were associated with an increase in the rate of collisions, resulting in injury and mortality.

Our study shows that there was a clear downward trend in the three outcomes studied, which initiated prior to the policy implemented in 2015. This trend has been attributed to several initiatives and improvements in road infrastructure in the city over the years.^6 28 29^ However, by 2015, Mexico City’s road traffic mortality rate was still substantially higher than other cities in the world at 8.7 per 100 000 compared, for example, to 3.7 per 100 000 in Buenos Aires or 1.6 per 100 000 in London.^30^ The city government announced a commitment to Vision Zero and designed and approved the new policies.^9 10^ While the effect of these policies was negligible in terms of overall collisions and collisions resulting in injury, they had the desired effect on mortality. Since the most important changes were speed limits and their enforcement with speed cameras and higher penalties, these results are consistent with prior literature. Lower speed limits have a stronger relationship to crash severity than to crashes overall.^31^ We did not find evidence that the speed cameras and other automated traffic enforcement devices modified the effect of other policy components. The regulations presumably had a more generalised effect on behaviours across the city as opposed to local to municipalities with cameras. New speed limits applied across the entire city and there was police enforcement using handheld devices in different points of the city.

The 2019 changes reverted the downward trend in collisions with injuries and mortality that had been observed until that point in time. These two outcomes originated from independent data sets, which strengthens this result. Changes in 2019 were announced in January and economic penalties stopped at that point; however, the new penalty system did not come into effect until June 2019 and its implementation is questionable. The change between 2018 and 2019 was drastic; in 2018, there were 4.3 million economic penalties associated with automated traffic enforcement devices, which dropped to zero in the first trimester of 2019 according to data available online from the city police department.^32 33^ Penalties picked up after April 2019, but the data suggest that only about 5% of^32^ violations ended up in a course or community service.^33^ Our results, therefore, may reflect that drivers became aware that there were no consequences for speeding and other road safety violations, and this may have resulted in more reckless driving behaviours. Evidence suggests that immediate feedback and certainty of penalties is even more important than their severity.^4^ Previous research also shows that police enforcement and economic penalties for traffic violations are effective at reducing their frequency. In one study in Austria, a penalty increase of 10 Euros was predicted to reduce the frequency of speeding by 5%.^14^ Conversely, a reduction in police citations for road safety violations in the province of Quebec, Canada was associated with an increase of eight additional collisions per month.^3^

The current Mexico City government has a challenge to correct course and prevent further road traffic deaths. The current regulations which rely on civic education, although well intentioned, have had a detrimental effect on health. Evidence-based, large-scale interventions are urgently needed and there are several policy options available. The government could revisit economic penalties linked to automated enforcement devices, leveraging the technology and systems that were already in place before 2019 but improving the programme’s weaknesses. For example, the equity aspect of the programme, which was a concern, could be addressed by sending warnings initially, before sending a fine. An alternative or accompanying intervention to economic penalties is designing roads to better manage and reduce vehicle speeds and other dangerous behaviours, such as narrowing travel lanes, reducing the number of travel lanes and speed feedback signs. Current and future governments need to ensure that regulations are well implemented and enforced.

One of the strengths of our study is that our analyses included three road safety outcomes that came from two unrelated databases showing consistent results. Furthermore, AXA information was collected at the site of the collision using a digital platform, which may ensure quality and comparability of the data across the city. Crucially, data collection is unlikely to have been affected by implementation of new road traffic regulations as opposed to data collected by police officers in the city (ie, police may have been more aware of road safety and more active reporting collisions). We conducted a series of sensitivity analyses, which broadly supported our main results. Although findings with redistributed mortality codes were more conservative, we use the original mortality data set for the main results for the following reasons: (1) estimates of deaths due to road injury in Mexico are considered reliable because the proportion of ill-defined and partially defined codes employed is less than 20%^34^ and (2) the process of redistributing codes includes several untestable assumptions, for example, codes may be redistributed incorrectly or the distribution of fully specified codes may not match the distribution of ill-defined and partially defined codes; therefore, redistributed mortality is also prone to error.

Our study also has some limitations. There tends to be a large under-registration of road traffic collisions, regardless of the data set used. AXA’s collision data represented only a subset of all collisions since just half of cars were insured in Mexico City and many insurance companies share the market. The implications of this are that we cannot draw conclusions on the rate of collisions in absolute terms or compare the rate to results from other data sources such as police reports. However, AXA data are appropriate to answer the question posed for this study because data collection over the period did not change. We make the assumption that AXA’s market share did not change either over the period of study in Mexico City; this assumption is supported by AXA’s financial reports^35^ and via personal communication with the Mexican Association of Insurance Companies. Including data from other insurance companies were not possible because they have not made it available for public use. A second limitation is that we are unable to isolate the effect of specific policy components, for example, speed limits, because several interventions came into effect at the same time. However, we show evidence for the package of interventions, which includes lower speed limits, enhanced enforcement and higher penalties especially compared with a period of looser enforcement and no economic penalties. A third limitation is that we were not able to measure intermediate outcomes, such as vehicle speeds before and after the policies. Finally, the distribution of automated traffic enforcement devices was limited to certain types of streets; this limits external validity of our analysis that investigated whether these devices modified the effect of policy changes.

In conclusion, this study supports city-level road safety regulations accompanied by enforcement with economic penalties. This is consistent with international recommendations, which note that strong enforcement of evidence-based recommendations can substantially improve road safety outcomes.^2^

## Data Availability

Data are available in a public, open access repository. Stata do-files are available upon reasonable request.
