# Supplementary material for: Evaluation of road safety policies and their enforcement in Mexico City, 2015–2019: an interrupted time-series study
Source: Inj Prev. 2022 Sep 12;29(1):35–41. doi: 10.1136/ip-2022-044590 (PMC7614109; doi:10.1136/ip-2022-044590)
Supplement: Supplementary data [file ip-2022-044590supp001.pdf]

BMJ Publishing Group Limited (BMJ) disclaims all liability and responsibility arising from any reliance placed on this supplemental material which has been supplied by the author(s)

Supplementary Material

Evaluation of road safety policies and their enforcement in Mexico City, 2015-2019: An Interrupted Time Series Study

Carolina Quintero Valverde, Carolina Pérez Ferrer, Luis Chías Becerril, Armando Martínez, Héctor Reséndiz Lopez, Javier Prado Galbarro, Alex Quistberg, Ana V. Diez-Roux, Tonatiuh Barrientos-Gutierrez

Table of contents for supplementary material

Figure S1. Location of automatic traffic enforcement devices in Mexico City, 2015 2
Data processing and cleaning 2
Table S1. Results of validation process 2
Figure S2. Collisions within and outside Mexico City 3
Equation 2. Controlled interrupted time series analyses 4
Table S2. Periods of analyses and number of data-points 5
Table S3. Effect of 2015 intervention on total collisions and collisions resulting in injury in enforcement and no-enforcement municipalities 5
Table S4. Total collisions, ITS CDMX, Sensitivity Analyses 6
Table S5. Collisions resulting in injury, ITS CDMX, Sensitivity Analyses 7
Table S6. Mortality due to road traffic collisions, ITS CDMX, Sensitivity Analyses 8
Figure S3. Interrupted Time Series of mortality with redistributed garbage codes in Mexico City. 9

Figure S1. Location of automatic traffic enforcement devices in Mexico City, 2015

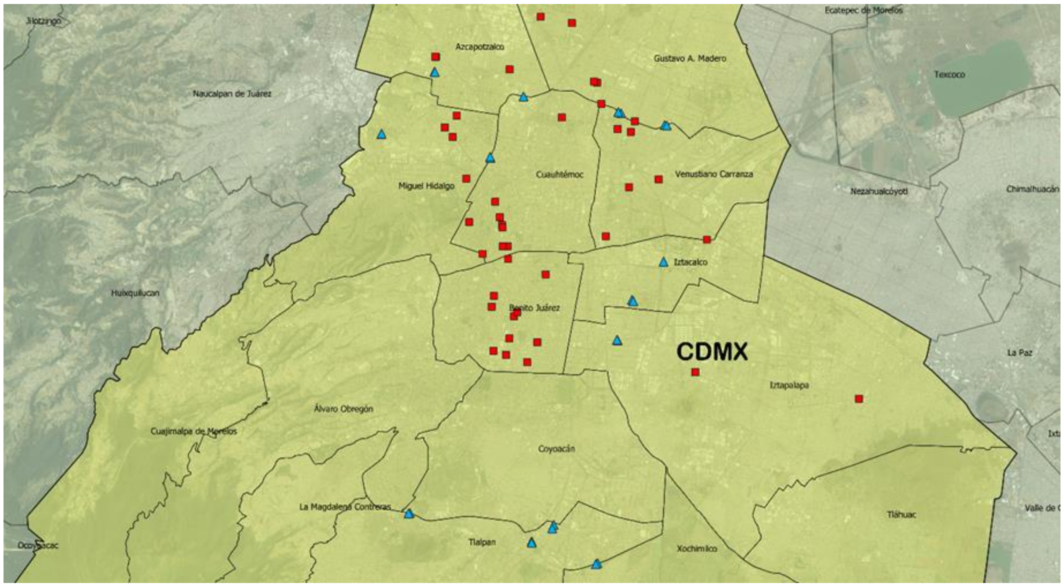

*Red squares are automatic traffic enforcement devices that detect 9 dangerous behaviors. Blue triangles are speed cameras. Original figure created by the research team.

Data processing and cleaning

Table S1. Results of validation process

Year	2015		2016		2017		2018		2019		Total	
	Total number	%	Total number	%	Total number	%	Total number	%	Total number	%	Total number	%
**Mexico City consistent in geographical coordinates and location variable**	87,406	91.0	84,889	90.0	74,159	91.9	65,496	91.4	55,237	92.6	367,187	91.3
**Mexico City consistent in geographical coordinates and inconsistent in location variable**	77	0.1	194	0.2	37	0.0	32	0.0	255	0.4	595	0.1
**Mexico City consistent in location variable and inconsistent in geographical coordinates**	8,556	8.9	9,193	9.8	6,502	8.1	6,151	8.6	4,194	7	34,596	8.6
**Mexico City Total**	96,039	100	94,276	100	80,698	100	71,679	100	59,681	100	402,378	100

Figure S2. Collisions within and outside Mexico City

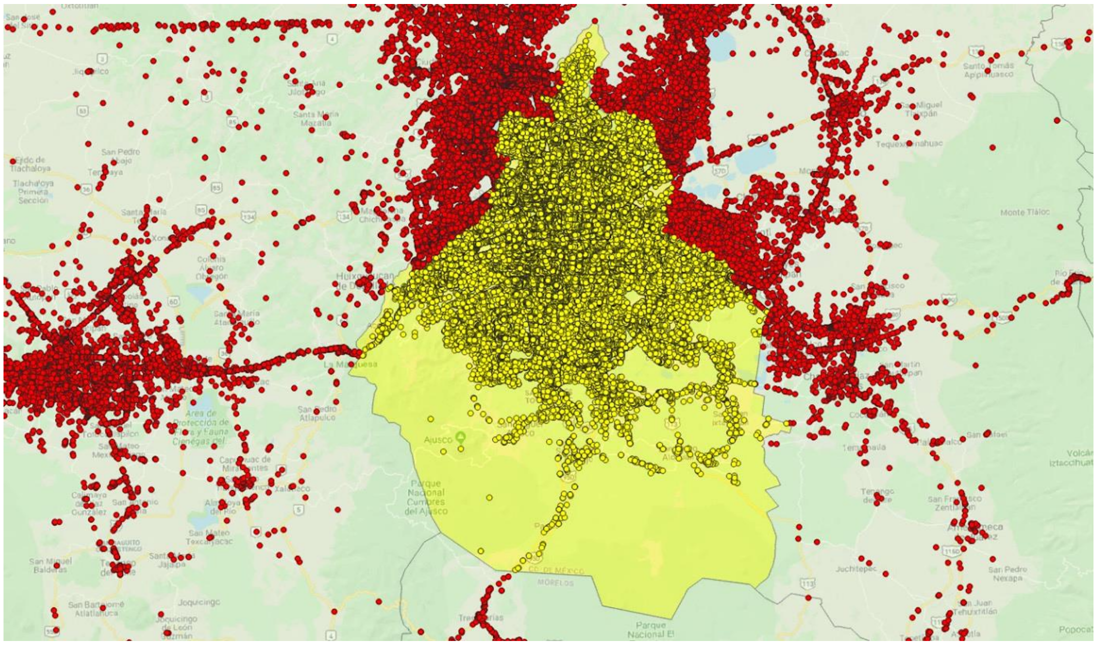

Note: Yellow polygon is Mexico City. Original figure created by the research team.

Equation 2. Controlled interrupted time series analyses

Equation 2.

$$Y_t = \beta_0 + \beta_1 T + \beta_2 X_t + \beta_3 TX_t + \beta_4 G + \beta_5 GT + \beta_6 GX_t + \beta_7 GTX_t$$

where Y_t is the outcome variable at time t, β_0 represents the intercept at T=0, β_1 is the change in outcome per time unit increase (representing the underlying pre-intervention trend), β_2 is the level change following the intervention and β_3 indicates the slope change following the intervention (using the interaction between time centered and intervention: TX_t) β_4 represents the difference in intercept at T=0 between enforcement and no enforcement municipalities, β_5 represents the difference in the time slope in municipalities with and without enforcement in the pre-intervention period, β_6 represents the difference in the level change associated with the intervention in enforcement compared to no-enforcement municipalities and β_7 represents the difference in the time slope following the intervention in enforcement compared to no-enforcement municipalities. These two parameters (β_6 and β_7) therefore capture whether enforcement modifies the effect of the intervention (on level β_6 and on the change in slope post intervention β_7). We checked for parallel pre-intervention trends between enforcement and no-enforcement municipalities by evaluating statistical significance of β_5.

Table S2. Periods of analyses and number of data-points

	Total collisions and collisions resulting in injury				Mortality			
	Pre-policy dates	Number of data-points*	Post-policy dates	Number of data-points*	Pre-policy dates	Number of data-points*	Post-policy dates	Number of data-points*
2015 policy	1st Jan 2015 - 14 Dec 2015	50	15 Dec 2015 - 31st Dec 2018	158	1st Jan 2013- 14 Dec 2015	154	15 Dec 2015 - 31st Dec 2018	158
2019 policy	15 Dec 2015- 7 June 2019	181	8 June 2019 - 31st Dec 2019	29	15 Dec 2015- 7 June 2019	181	8 June 2019 - 31st Dec 2019	29

* weeks

Table S3. Effect of 2015 policy on total collisions and collisions resulting in injury in enforcement and no-enforcement municipalities

	Total collisions			Collisions resulting in injury		
	IRR	P	95%CI	IRR	P	95%CI
Time (β1)	1.000	0.627	0.998 1.003	0.998	0.472	0.994 1.003
Intervention (β2)	1.058	0.228	0.965 1.160	1.066	0.401	0.919 1.237
Time X intervention (β3)	0.996	0.004	0.993 0.999	0.997	0.169	0.993 1.001
Enforcement (β4)	1.044	0.447	0.934 1.168	1.043	0.641	0.872 1.248
Enforcement X time (β5)	0.999	0.975	0.996 1.004	0.999	0.832	0.993 1.005
**Enforcement X intervention (β6)**	**0.957**	**0.510**	**0.840 1.091**	**0.959**	**0.691**	**0.779 1.180**
**Enforcement X time X intervention (β7)**	**0.999**	**0.995**	**0.996 1.004**	**1.001**	**0.641**	**0.995 1.008**

*In bold, coefficients of interest to test the hypothesis of a difference in level changes between enforcement and no-enforcement municipalities (**β6), and difference in** slope differences between enforcement and no-enforcement municipalities (**β7**)

Table S4. Total collisions, ITS CDMX, Sensitivity Analyses

	Main analyses			Gasoline shortage			Private vehicles selected			2015 Intervention moved to June 2016		
	IRR	p	95%CI	IRR	p	95%CI	IRR	p	95%CI	IRR	p	95%CI
**2015**												
Step level change 2015	1.057	0.197	0.971, 1.151				1.004	0.931	0.921, 1.094	1.053	0.142	0.983, 1.128
Pre-2015 trend (Jan-Dec 2015)	0.998	0.084	0.995, 1.000				0.998	0.234	0.996, 1.001	0.998	0.001	0.997, 0.999
Post-2015 trend (Jan 2016-Dec 2018)	0.997	0.000	0.997, 0.997				0.998	0.000	0.997, 0.998	0.997	0.000	0.996, 0.997
Slope difference – 2015 intervention	0.999	0.536	0.997, 1.002				0.999	0.636	0.997, 1.002	0.999	0.052	0.997, 1.000
**2019**												
Step level change 2019	1.028	0.600	0.927, 1.140	1.024	0.652	0.924, 1.134						
Pre-2019 trend (Jan 2016 – 7 June 2019)	0.997	0.000	0.997, 0.997	0.997	0.000	0.997, 0.997						
Post-2019 trend (8 June 2019 - 31st Dec 2019)	0.997	0.358	0.991, 1.003	0.997	0.341	0.991, 1.003						
Slope difference – 2019 intervention	1.000	0.973	0.994, 1.006	1.000	0.994	0.994, 1.006						

Table S5. Collisions resulting in injury, ITS CDMX, Sensitivity Analyses

	Main analyses			Gasoline shortage			Private vehicles selected			2015 Intervention moved June 2016		
	IRR	p	95%CI	IRR	p	95%CI	IRR	p	95%CI	IRR	p	95%CI
**2015**												
Step level change 2015	1.031	0.595	0.922, 1.153				1.009	0.879	0.901, 1.130	1.091	0.059	0.997, 1.195
Pre-2015 trend (Jan-Dec 2015)	0.996	0.019	0.993, 0.999				0.996	0.018	0.993, 0.999	0.996	0.000	0.994, 0.998
Post-2015 trend (Jan 2016-Dec 2018)	0.997	0.000	0.996, 0.997				0.997	0.000	0.997, 0.998	0.996	0.000	0.995, 0.997
Slope difference – 2015 intervention	1.000	0.795	0.997, 1.004				1.001	0.523	0.998, 1.004	1.000	0.952	0.998, 1.002
**2019**												
Step level change 2019	1.066	0.435	0.909, 1.250	1.074	0.373	0.918, 1.258						
Pre-2019 trend (Jan 2016 – 7 June 2019)	0.996	0.000	0.996, 0.997	0.996	0.000	0.996, 0.997						
Post-2019 trend (8 June 2019 - 31st Dec 2019)	1.011	0.028	1.001, 1.021	1.011	0.032	1.001, 1.020						
Slope difference – 2019 intervention	1.015	0.003	1.005, 1.025	1.014	0.003	1.005, 1.024						

Table S6. Mortality due to road traffic collisions, ITS CDMX, Sensitivity Analyses

	Main Analyses			Gasoline shortage			Private vehicles selected			2015 Intervention moved to June 2016			Redistribution of garbage codes		
	IRR	p	95%CI	IRR	p	95%CI	IRR	p	95%CI	IRR	p	95%CI	IRR	p	95%CI
2015															
Step level change 2015	0.960	0.580	0.829, 1.111				1.016	0.910	0.765, 1.351	1.003	0.971	0.863, 1.165	0.926	0.169	0.831, 1.033
Pre-2015 trend (Jan-Dec 2015)	0.999	0.099	0.998, 1.000				0.997	0.002	0.994, 0.999	0.999	0.003	0.998, 1.000	0.999	0.226	0.999, 1.000
Post-2015 trend (Jan 2016-Dec 2018)	0.997	0.000	0.996, 0.998				0.994	0.000	0.992, 0.997	0.997	0.000	0.995, 0.998	1.000	0.297	0.999, 1.000
Slope difference – 2015 intervention	0.998	0.038	0.997, 1.000				0.998	0.177	0.995, 1.001	0.998	0.058	0.996, 1.000	1.000	0.910	0.999, 1.001
2019															
Step level change 2019	0.788	0.133	0.577, 1.075	0.774	0.105	0.568, 1.055							0.958	0.684	0.780, 1.177
Pre-2019 trend (Jan 2016 – 7 June 2019)	0.996	0.000	0.995, 0.997	0.996	0.000	0.995, 0.997							0.999	0.013	0.998, 1.000
Post-2019 trend (8 June 2019 -31st Dec 2019)	1.023	0.008	1.006, 1.041	1.023	0.008	1.006, 1.041							1.002	0.802	0.990, 1.013
Slope difference – 2019 intervention	1.027	0.002	1.010, 1.045	1.027	0.002	1.010, 1.045							1.002	0.689	0.991, 1.014

Figure S3. Interrupted Time Series of mortality with redistributed garbage codes in Mexico City.

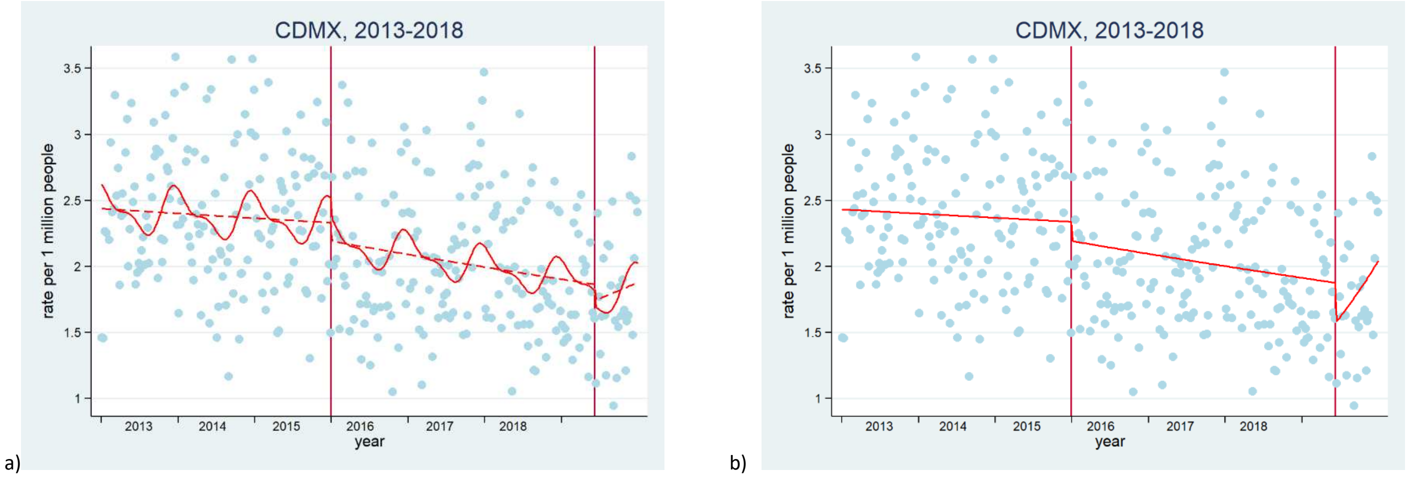

Blue dots=estimated road traffic deaths after redistribution in Mexico City. Continuous lines=trends. Vertical lines: delineate the interventions in December 2015 and June 2019. Panel a. model that adjusts for seasonality (consistent with table S6), Panel b. model without adjusting for seasonality (consistent with main analyses). Original figures created by the research team.
